# Differences in colonoscopy performance among four endoscopy centers in Western Norway: Influence of case-mix

**DOI:** 10.1055/a-2546-9515

**Published:** 2025-04-04

**Authors:** Tom Andre Pedersen, Trond Engjom, Georg Gjorgji Dimcevski, Edoardo Botteri, Birgitte Seip, Roald Flesland Havre

**Affiliations:** 1 60498 Department of Medicine, Haukeland University Hospital, Bergen, Norway; 2 72982 Department of Medicine, Haraldsplass Deaconess Hospital, Bergen, Norway; 3 1658 Bergen Research group for Advanced Gastrointestinal Endoscopy (BRAGE), Department of Clinical Medicine, University of Bergen, Bergen, Norway; 4 1658 Department of Clinical Medicine, University of Bergen, Bergen, Norway; 5 Private outpatient endoscopy centre, Kanalspesialistene AS, Bergen, Norway; 6 11315 Section for Colorectal Cancer screening, Cancer Registry of Norway, Oslo, Norway; 7 11315 Department of Research, Cancer Registry of Norway, Oslo, Norway; 8 60512 Department of Medicine, Vestfold Hospital Trust, Tonsberg, Norway

**Keywords:** Endoscopy Lower GI Tract, Polyps / adenomas / ..., Colorectal cancer, CRC screening, Quality and logistical aspects, Sedation and monitoring, Quality management

## Abstract

**Background and study aims:**

Unmodifiable patient factors such as age, sex, and indication (case-mix) may influence colonoscopy performance. In this study, we explored how case-mix affected polyp detection, cecal intubation, and pain on a center level.

**Methods:**

A cross-sectional study was performed on data from four centers in Western Norway registered in the national endoscopy quality registry, Gastronet, in 2020 and 2021. We extracted demographics, indication, and the performance measures cecal intubation rate (CIR), proportion of at least one polyp ≥ 5 mm in size per colonoscopy (PDR-5), and pain. We also analyzed the explanatory variables bowel preparation, withdrawal time, and sedation/analgesia.

**Results:**

First colonoscopies in 14,765 patients were included. Median age was 60 years (interquartile range 46–71) and 54% were women. Case-mix differed between centers and significantly influenced performance measures. Increased PDR-5 was associated with higher age and male sex (odds ratio [OR] 1.27, 95% confidence interval [CI] 1.18–1.37). The indication surveillance had the highest PDR-5 (44.9%, 95% CI 42.6–47.1) and inflammatory bowel disease the lowest (14.6%, 95% CI 12.3–16.8). CIR decreased with increasing age. Men had less pain (OR 0.33, 95% CI 0.27–0.39). Among indications, surveillance and IBD had higher CIRs and less pain. Performance measures differed among centers, even after adjustment for case-mix and other known explanatory variables such as sedation/analgesia and bowel preparation.

**Conclusions:**

Case-mix influenced performance measures. Although we showed center differences in performance, other factors, such as individual endoscopist skills, probably influence performance measures. Our study demonstrates the importance of considering case-mix when assessing colonoscopy performance.

## Introduction

Colorectal cancer (CRC) was diagnosed in 1.9 million people worldwide in 2022, making it the third most common cancer. Norway has the second highest incidence of CRC in the world after Denmark, with an estimated age-standardized incidence rate of 45.3 per 100,000 population 1 . Studies have shown that an increased adenoma detection rate (ADR) reduces risk of post-colonoscopy CRC 2 3 .

In 2017, the European Society of Gastrointestinal Endoscopy (ESGE) published performance measures for lower gastrointestinal endoscopy as a quality improvement initiative 4 . In Norway, the performance measures are registered in Gastronet 5 , the national quality registry for colonoscopy. Gastronet uses PDR-5 (the proportion of at least one polyp ≥ 5 mm in size per colonoscopy) as a surrogate marker for ADR. High-quality colonoscopies are a prerequisite for preventing CRC.

Numerous factors can influence colonoscopy performance 4 6 7 8 . When seeking to improve colonoscopy quality, the focus has been mainly on factors related to the endoscopist and bowel preparation. However, unmodifiable patient factors such as age, sex, and indication for colonoscopy, the case-mix, may vary between institutions and explain performance differences 9 10 11 12 .

Regarding earlier studies on case-mix factors and quality indicators, Corley et al. demonstrated that older and male patients have more adenomas 13 . Boroff et al. showed that the indication colitis had relatively low ADR 7 . Several studies showed that endoscopies performed as surveillance after polypectomy or CRC treatment had high ADR 6 7 8 . Seip et al. 14 and Holme et al. 15 showed that pain was associated with female sex and age < 40 years. Harris et al. showed considerable variations in colonoscopy performance between centers. Controlling for case-mix did not significantly modify these variations, indicating that center and procedure characteristics play a role 16 .

In a real-life patient mix, including inflammatory bowel disease (IBD), variances in pretest probability for presence of polyps created by differences in population case-mix may influence PDR-5. Furthermore, case-mix factors may also impact the other colonoscopy quality parameters cecal intubation rate (CIR) and severe pain. In this study, based on endoscopist and patient-reported data in Gastronet, we aimed to: 1) describe differences in patient characteristics and indications for colonoscopy among four colonoscopy centers covering a population of approximately 400,000; and 2) identify how differences in case-mix influence colonoscopy performance on a center level.

## Patients and methods

### Study design and subjects

We performed a cross-sectional study of data collected in Gastronet from January 1, 2020 to December 31, 2021 about patients who underwent colonoscopy at a university clinic (Center 1), two local hospitals (Centers 2 and 4), and a high-volume outpatient endoscopy practice (Center 3). The Western Norway Regional Health Authority finances all centers and has public health responsibility for a population of approximately 400,000. The university clinic is a public, academic hospital with a substantial cohort of IBD patients. The two local hospitals are smaller public hospitals with fewer IBD patients. The three hospitals have endoscopy trainees, whereas the outpatient endoscopy center only has experienced endoscopists. The outpatient endoscopy center is private and has a public revenue agreement. It has few IBD patients. Two hospitals are CRC screening centers, but the national screening program did not start during the study period. The few cases classified as “screening” refer to surveillance colonoscopies in families with CRC or “wild screening.”

**Fig. 1:**
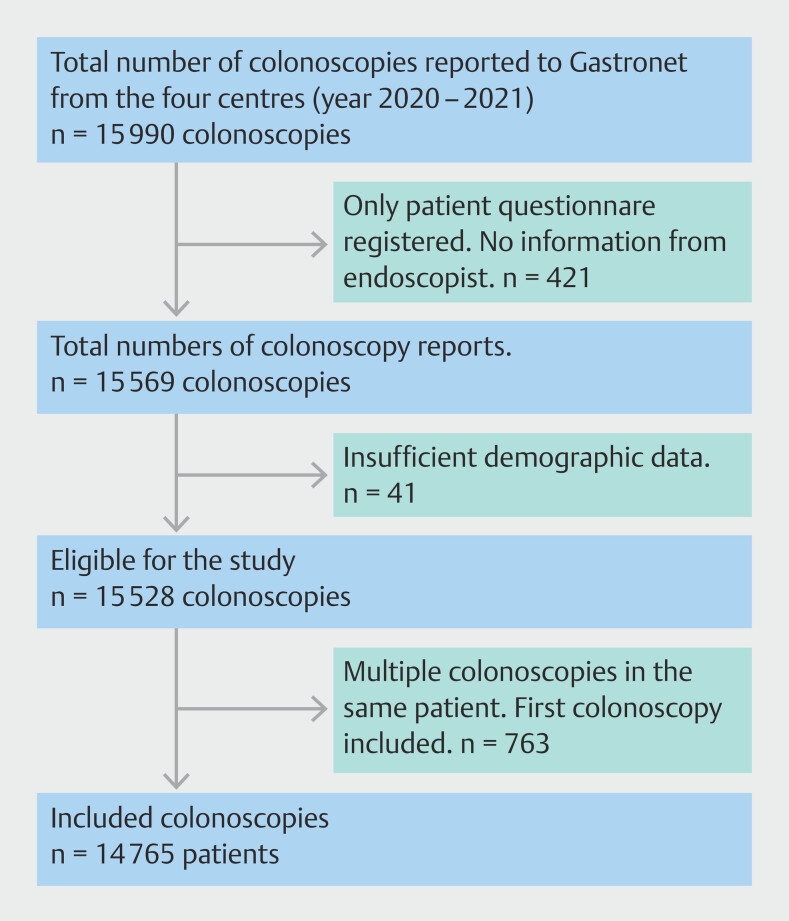
Patient inclusion.

Gastronet receives a report from the endoscopist and the patient. Colonoscopies for which only the patient questionnaire was received and not the endoscopist questionnaire were excluded. Colonoscopies with inadequate or missing demographic data were also excluded. In patients with more than one colonoscopy performed during the study period, we only included the first colonoscopy ( Fig. 1 ).

### Data collection from Gastronet

Gastronet data are acquired from a structured endoscopy report filled in by the endoscopist and a patient-reported outcome measures (PROM) questionnaire completed by the patient after the examination.

We extracted the following variables from Gastronet: demographic data (age, sex), indication for colonoscopy, use of sedation/analgesia, Boston Bowel Preparation Scale (BBPS), CIR (unadjusted), PDR-5, withdrawal time, and patient-reported pain (no, some, moderate, or severe). Colonoscopy indications registered in Gastronet are several: symptoms, post-polypectomy surveillance, post-CRC surveillance, family history of CRC, screening, IBD, diverticulitis, appendicitis, or other. Due to few examinations for some of the indications, we combined them into the following categories: Symptoms, surveillance (post-polypectomy/-CRC), IBD, CRC family/screening and other (diverticulitis, appendicitis, and other).

Recommended standards of performance measures in Gastronet are based on diagnostic and surveillance indications and include PDR-5 ≥25% in patients ≥50 years, CIR ≥90%, and severe pain ≤15%. Colonoscopies are performed without or with moderate sedation/analgesia, commonly midazolam and/or alfentanil administered intravenously 17 . Doses of midazolam doses are typically 1 to 2 mg and of alfentanil are 0.25 to 0.50 mg. Self-reported data by the endoscopists include indication for colonoscopy, use of sedation/analgesia with doses, bowel preparation (BBPS) 18 ), CIR (unadjusted for poor bowel preparation or not passable strictures), PDR-5, and withdrawal time (WT). In Gastronet the registered WT is the time spent inspecting the mucosa during withdrawal of the colonoscope from the cecum to the anal canal, rounded to the nearest whole minute, also including polypectomies and/or biopsies if performed. Gastronet uses PDR-5 as a surrogate marker for ADR 5 19 . The endoscopist estimates polyp size using a biopsy forceps or polypectomy snare as a reference. PDR-5 is readily available, not dependent on histology and encompasses both adenomas and serrated polyps.

In Norway, all clinical activity is reported to the Norwegian Patient Register (NPR). Completeness of NPR is considered to be 100% because non-reported procedures are unpaid. NPR is used to estimate coverage of reporting to Gastronet. Completeness in this study was defined as the proportion of NPR-registered outpatient colonoscopies reported to Gastronet in 2020 and 2021.

### Ethical considerations

Gastronet uses an anonymous registration number linked to the patient, endoscopist, and center. Information in Gastronet is collected according to The Norwegian Directorate of Health’s exemption from the duty of confidentiality. The Norwegian Data Protection Authority licenses the register. The concession does not require patient consent for registration, but patients have the right to opt out of registration and are informed how to do this in writing 20 . The Regional Committee for Medical and Health Research Ethics (REC) of Western Norway approved the study (REC-identification number 275068).

### Statistical analyses

Continuous variables were presented as medians with interquartile ranges (IQRs). Categorical variables were presented as frequencies and percentages with 95% confidence interval (CI) using the Wald method. Pearson’s chi-squared test was used to test differences for categorical variables. Student’s *t* -test was used to evaluate continuous variables. Univariable and multivariable logistic regression analyses were performed to estimate the effect of the case-mix factors and center on the three performance measures. We reported odds ratio (OR) and 95% CI. We performed a sensitivity analysis in which we also included BBPS and sedation in the multivariable models. Withdrawal time was left out of the multivariable regression analyses because of a large amount of excluded data, because we only included colonoscopies without biopsies or interventions. Withdrawal time is essential for visualizing as much mucosa as possible; however, total withdrawal time may not reflect mucosa visualization when polypectomies or multiple biopsies are performed. Statistical significance was defined as *P* <0.05 using two-sided tests. Data were analyzed using IBM SPSS Statistics Version 29 (IBM, Armonk, New York, United States).

## Results

### Inclusion

A total of 15,990 colonoscopies were reported to Gastronet during the study period and 1225 examinations were excluded ( Fig. 1 ). A total of 14,765 colonoscopies/patients were included and 7873 patient questionnaires (53.3%) were returned.

### Coverage of Gastronet

In 2020 and 2021, coverage of colonoscopies reported to Gastronet increased from 71.1% to 79% in Center 1 but decreased in all the other clinics from 69.5% to 62.3% in Center 2, 93.9% to 82.9% in Center 3, and 93.8% to 92.7% in Center 4. The proportion of returned patient questionnaires was 47.3% (935/1978) for Center 1, 56.8% (676/1190) for Center 2, 52.7% (5481/10410) for Center 3, and 65.8% (781/1187) for Center 4.

### Case-mix

Table 1 shows case-mix characteristics of each center. There was significant sex difference; 54% were women ( *P* = 0.002). Median age of the total study population was 60 years (IQR 46–71). There were age differences between the center populations; Center 4 had the highest age at median 65 years (IQR 53–73) and Center 1 the lowest at median 55 years (IQR 38–68), *P* < 0.001. Indication frequency varied significantly among centers, *P* < 0.001, e.g. IBD was the indication for colonoscopy in 31.2% of the patients at Center 1 and 1.3% at Center 3.

**Table:** **Table 1** Patient and center characteristics (case-mix per center).

	Total N (%)	Center 1 N (%)	Center 2 N (%)	Center 3 N (%)	Center 4 N (%)	*P* value
Patients	14765 (100)	1978 (100)	1190 (100)	10410 (100)	1187 (100)	
Sex						0.002
Men	6797 (46.0)	936 (47.3)	594 (49.9)	4693 (45.1)	574 (48.4)	
Women	7968 (54.0)	1042 (52.7)	596 (50.1)	5717 (54.9)	613 (51.6)	
Age, years						< 0.001
< 40	2518 (17.1)	542 (27.4)	230 (19.3)	1615 (15.5)	131 (11.0)	
40–49	2003 (13.6)	289 (14.6)	155 (13.0)	1445 (13.9)	114 (9.6)	
50–59	2745 (18.6)	336 (17.0)	187 (15.7)	2017 (19.4)	205 (17.3)	
60–69	3343 (22.6)	370 (18.7)	235 (19.7)	2448 (23.5)	295 (24.9)	
70–79	3147 (21.3)	344 (17.4)	277 (23.3)	2206 (21.2)	320 (27.0)	
≥ 80	1004 (6.8)	97 (4.9)	106 (8.9)	679 (6.5)	122 (10.3)	
Median (IQR)	60 (46–71)	55 (38–68)	61 (44–72)	60 (47–71)	65 (53–73)	
Indications						<0.001
Symptoms	8860 (60.4)	804 (41.3)	707 (60.3)	6608 (63.6)	741 (63.4)	
Surveillance (polyp, CRC)	1879 (12.8)	226 (11.6)	205 (17.5)	1267 (12.2)	181 (15.5)	
CRC family/screening	1343 (9.2)	161 (8.3)	47 (4.0)	1070 (10.3)	65 (5.6)	
IBD	941 (6.4)	607 (31.2)	129 (11.0)	135 (1.3)	70 (6.0)	
Other	1649 (11.2)	149 (7.7)	84 (7.2)	1305 (12.6)	111 (9.5)	
Missing	93	31	18	25	19	
CRC, colorectal cancer; IBD, inflammatory bowel disease; IQR, interquartile range. Center 1: University clinic. Centers 2 and 4: Local hospitals. Center 3: Outpatient endoscopy practice.						

### Performance measures

**Fig. 2:**
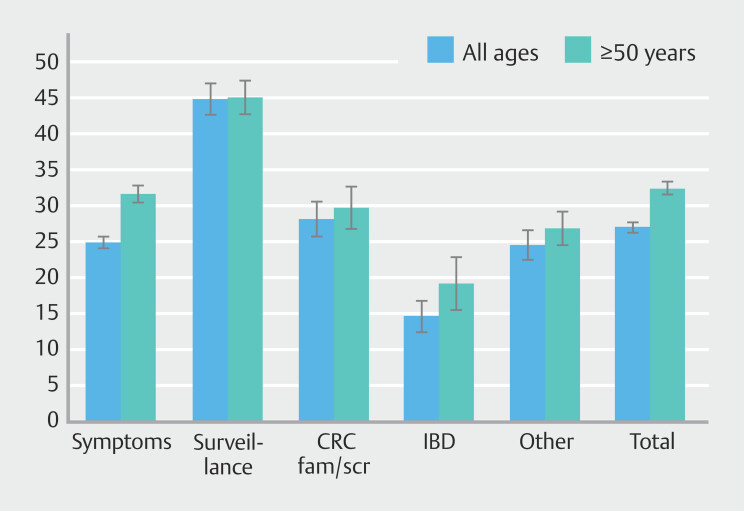
PDR-5 per indication (% with 95 % confidence interval), all centers.

PDR-5 per indication varied substantially, as illustrated in Fig. 2 . The highest PDR-5 was found in surveillance colonoscopies in patients aged ≥ 50 years at 45.1% and the lowest in IBD patients at 19.1% ( *P* < 0.001).

**Fig. 3:**
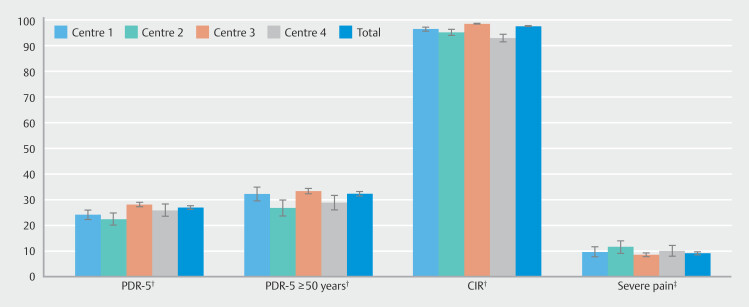
Performance measures per center (%) with 95% confidence interval. Significant differences between centres: †p <0.001. ‡p=0.042.

**Fig. 4:**
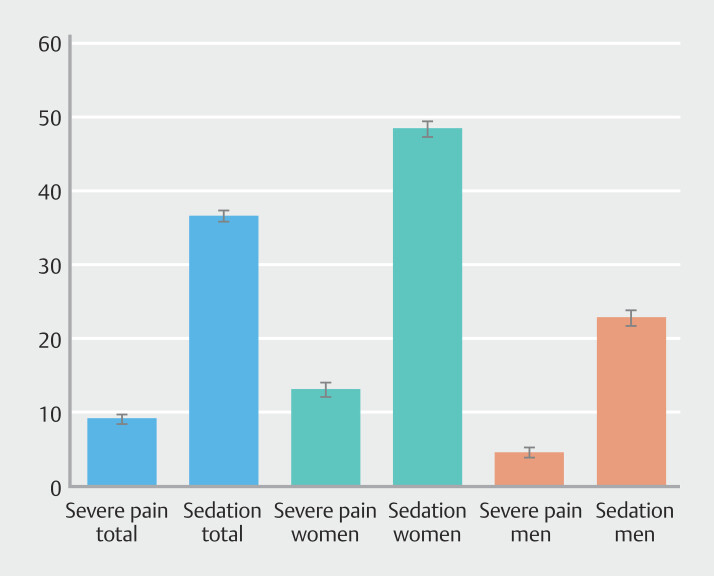
Severe pain and sedation/analgesia. % with 95 % confidence interval. Women report more severe pain (13.1%) than men (4.6%) (p<0.001). More women (48.3%) than men (22.8%) receive sedation/analgesia (p<0.001).

Table 2 shows differences in performance measures among the centers. Severe pain was reported in < 15% of examinations at all centers, but with significant center differences ( *P* = 0.042) ( Table 2 and Fig. 3 ). Sedation/analgesia was administered more frequently to women than to men ( *P* < 0.001). Regardless of this, in all centers, women reported significantly more pain than men ( *P* < 0.001) ( Fig. 4 ). Withdrawal time (without biopsies or intervention) was significantly different among the centers, with Center 1 having the longest of 10 minutes (IQR 7–12) and Center 2 the shortest of 7 minutes (IQR 5–10) ( *P* < 0.001).

**Table:** **Table 2** Performance measures per center.

	Total N (% [95%CI])	Center 1 N (% [95%CI])	Center 2 N (% [95%CI])	Center 3 N (% [95%CI])	Center 4 N (% [95%CI])	*P* value
PDR-5	3988 (27.0 [26.3–27.7])	479 (24.2 [22.3–26.1])	268 (22.5 [20.1–24.9])	2933 (28.2 [27.3–29.0])	308 (25.9 [23.5–28.4])	< 0.001
PDR-5 ≥ 50 years	3316 (32.4 [31.5–33.3])	370 (32.3 [29.6–35.0])	216 (26.8 [23.8–29.9])	2458 (33.4 [32.4–34.5])	272 (28.9 [26.0–31.8])	< 0.001
CIR						< 0.001
Yes	14278 (97.6 [97.4–97.8])	1856 (96.5 [95.6–97.3])	1116 (95.2 [94.0–96.4])	10228 (98.6 [98.4–98.8])	1078 (93.0 [91.5–94.5])	
No	349 (2.4 [2.1–2.4])	68 (3.5 [2.7–4.4])	56 (4.8 [3.6–6.0])	144 (1.4 [1.2–1.6])	81 (7.0 [5.5–8.5])	
Missing	138	54	18	38	28	
BBPS						< 0.001
≥ 6	13514 (96.0 [95.7–96.3])	1717 (92.9 [91.7–94.0])	1039 (94.9 [93.6–96.2])	9750 (97.0 [96.6–97.3])	1008 (93.2 [91.7–94.7])	
< 6	566 (4.0 [3.7–4.3])	132 (7.1 [6.0–8.3])	56 (5.1 [3.8–6.4])	304 (3.0 [2.7–3.4])	74 (6.8 [5.3–8.3])	
Missing	685	129	95	356	105	
Pain, severe						0.042
Yes	713 (9.2 [8.5–9.8])	90 (9.7 [7.8–11.7])	78 (11.7 [9.2–14.1])	467 (8.6 [7.9–9.4])	78 (10.1 [8.0–12.3])	
No	7072 (90.8 [90.2–91.5])	834 (90.3 [88.3–92.2])	591 (88.3 [85.9–90.8])	4955 (91.4 [90.6–92.1])	692 (89.9 [87.7–92.0])	
Missing	6980	1054	521	4988	417	
Sedation/analgesia						< 0.001
Yes	5360 (36.8 [36.1–37.6)])	1286 (69.0 [66.9–71.1])	581 (50.7 [47.8–53.5])	2664 (25.7 [24.8–26.5])	829 (71.5 [68.9–74.1])	
No	9190 (63.2 [62.4–63.9])	579 (31.0 [28.9–33.1])	566 (49.3 [46.5–52.2])	7714 (74.3 [73.5–75.2])	331 (28.5 [25.9–31.1])	
Missing	215	113	43	32	27	
Withdrawal time*						< 0.001
≥ 6 min	4093 (75.2 [74.1–76.4])	317 (87.1 [83.6–90.5])	267 (63.0 [58.4–67.6])	3126 (74.9 [73.6–76.2])	383 (79.8 [76.2–83.4])	
< 6 min	1347 (24.8 [23.6–25.9])	47 (12.9 [9.5–16.4])	157 (37.0 [32.4–41.6])	1046 (25.1 [23.8–26.4])	97 (20.2 [16.6–23.8])	
Excluded	9325	1614	766	6238	707	
Median (IQR)	8 (6–10)	10 (7–12)	7 (5–10)	8 (5–11)	8 (6–10)	
BBPS, Boston Bowel Preparation Scale; CIR, cecal intubation rate; IQR, interquartile range; PDR-5, proportion of at least one polyp ≥ 5 mm in size per colonoscopy. Center 1: University clinic. Centers 2 and 4: Local hospitals. Center 3: Outpatient endoscopy practice. *Without biopsies/therapy.						

### Case-mix influence on performance measures

Table 3 displays univariable and multivariable regression analyses of the performance measures PDR-5, CIR, and severe pain related to the case-mix (sex, age, indication) and center.

**Table:** **Table 3** Univariable and multivariable analyses of case-mix and performance measures.

		PDR-5			CIR			Severe pain		
Variable	Categories	N N (% [95% CI])	*P* value	OR (95 % CI)	N N (% [95% CI])	*P* value	OR (95 % CI)	N N (% [95% CI])	*P* value	OR (95 % CI)
**Sex**	Women	1959 (24.6 [23.6–25.5])	**< 0.001**	Reference	7694 (97.4 [97.1–97.8])	0.140	Reference	549 (13.1 [12.1–14.1])	**< 0.001**	Reference
	Men	2029 (29.9 [28.8–30.9])		**1.27 (1.18–1.37)**	6584 (97.8 [97.5–98.2])		1.21 (0.97–1.51)	164 (4.6 [3.9–5.2])		**0.33 (0.27–0.39)**
**Age, years**	< 40	280 (11.1 [9.9–12.3])	**< 0.001**	Reference	2465 (98.9 [98.5–99.3])	**< 0.001**	Reference	71 (11.4 [8.9–13.9])	0.290	Reference
	40–49	392 (19.6 [17.8–21.3])		**1.85 (1.57–2.19)**	1969 (98.9 [98.4–99.4])		0.87 (0.49–1.54)	58 (8.2 [6.2–10.3])		0.71 (0.49–1.03)
	50–59	627 (22.8 [21.3–24.4])		**2.25 (1.93–2.63)**	2670 (98.1 [97.5–98.6])		**0.48 (0.30–0.77)**	134 (9.5 [8.0–11.1])		0.87 (0.63–1.19)
	60–69	1198 (35.8 [34.2–37.4])		**3.96 (3.42–4.58)**	3241 (97.5 [97.0–98.1])		**0.37 (0.24–0.59)**	184 (8.5 [7.3–9.7])		0.80 (0.59–1.08)
	70–79	1136 (36.1 [34.4–37.8])		**4.03 (3.47–4.67)**	2988 (96.2 [95.5–96.8])		**0.26 (0.17–0.40)**	210 (9.4 [8.2–10.6])		0.87 (0.65–1.17)
	≥ 80	355 (35.4 [32.4–38.3])		**4.01 (3.34–4.82)**	945 (95.4 [94.0–96.7])		**0.23 (0.14–0.38)**	56 (8.6 [6.4–10.7])		0.75 (0.51–1.10)
**Indications**	Symptoms	2206 (24.9 [24.0–25.8])	**< 0.001**	Reference	8573 (97.5 [97.1–97.8])	**< 0.001**	Reference	447 (10.1 [9.2–11.0])	**< 0.001**	Reference
	Surveillance (polyp, CRC)	843 (44.9 [42.6–47.1])		**1.88 (1.69–2.09)**	1834 (98.1 [97.5–98.7])		**1.90 (1.31–2.74)**	73 (6.1 [4.7–7.4])		**0.66 (0.51–0.86)**
	CRC family/screening	377 (28.1 [25.7–30.5])		1.12 (0.98–1.28)	1320 (99.0 [98.5–99.6])		**2.27 (1.29–4.02)**	53 (6.9 [5.1–8.7])		**0.69 (0.51–0.93)**
	IBD	137 (14.6 [12.3–16.8])		**0.64 (0.52–0.78)**	903 (98.0 [97.2–98.9])		**1.74 (1.04–2.92)**	25 (5.9 [3.6–8.1])		**0.52 (0.33–0.81)**
	Other	404 (24.5 [22.4–26.6])		**0.79 (0.70–0.89)**	1578 (96.9 [96.1–97.8])		0.88 (0.64–1.20)	112 (12.3 [10.2–14.4])		**1.34 (1.07–1.68)**
	Missing	21 (22.6 [14.1–31.1])		0.89 (0.54–1.46)	70 (88.6 [81.6–95.6])		**0.34 (0.16–0.71)**	3 (5.7 [0–11.9])		0.52 (0.16–1.71)
**Center**	Center 1	479 (24.2 [22.3–26.1])	**< 0.001**	1.02 (0.90–1.15)	1856 (96.5 [95.6–97.3])	**< 0.001**	**0.30 (0.22–0.41)**	90 (9.7 [7.8–11.7])	**0.040**	**1.36 (1.05–1.76)**
	Center 2	268 (22.5 [20.1–24.9])		**0.72 (0.62–0.83)**	1116 (95.2 [94.0–96.4])		**0.27 (0.20–0.37)**	78 (11.7 [9.2–14.1])		**1.57 (1.21–2.04)**
	Center 3	2933 (28.2 [27.3–29.0])		Reference	10228 (98.6 [98.4–98.8])		Reference	467 (8.6 [7.9–9.4])		Reference
	Center 4	308 (25.9 [23.5–28.4])		**0.79 (0.69–0.91)**	1078 (93.0 [91.5–94.5])		**0.21 (0.16–0.28)**	78 (10.1 [8.0–12.3])		1.26 (0.98–1.64)
CI, confidence interval; CIR, cecal intubation rate; CRC, colorectal cancer; IBD, inflammatory bowel disease; OR, odds ratio PDR-5, proportion of at least one polyp ≥ 5 mm in size per colonoscopy. Bold font identifies significant results. Center 1: University clinic. Centers 2 and 4: Local hospitals. Center 3: Outpatient endoscopy practice. *P* values derive from Pearson’s chi-squared test (univariable analysis). ORs were calculated through a binary logistic regression (multivariable analysis).										

PDR-5 increased with male sex (OR 1.27, 95 % CI 1.18–1.37) and higher age. PDR-5 was significantly influenced by indication, i.e. surveillance (OR 1.88, 95% CI 1.69–2.09) and IBD (OR 0.64, 95% CI 0.52–0.78). Center influenced PDR-5, with Center 2 (OR 0.72, 95% CI 0.62–0.83) and Center 4 (OR 0.79, 95% CI 0.69–0.91) having significantly lower PDR-5.

CIR was reduced by increasing age. CIR was significantly influenced by indication, i.e. surveillance (OR 1.90, 95% CI 1.31–2.74) and IBD (OR 1.74, 95% CI 1.04–2.92). CIR was not affected by sex (OR 1.21, 95% CI 0.97–1.51).

Severe pain was less frequent in men (OR 0.33, 95% CI 0.27–0.39). Indication significantly influenced pain, i.e. surveillance (OR 0.66, 95% CI 0.51–0.86) and IBD (OR 0.52, 95% CI 0.33–0.81). Severe pain was not affected by age. There was a significant difference in severe pain when comparing the age groups over and under 40 years with univariable analysis. In the group aged < 40 years, 71 of 622 patients (11.4%, 95% CI 8.9–13.9) reported severe pain, whereas 642 of 7163 patients aged ≥ 40 years (9.0%, 95% CI 8.3–9.6) reported severe pain ( *P* = 0.042). When performing multivariable regression analysis with the case-mix factors, age < 40 years was not significant for severe pain (OR 1.22, 95% CI 0.93–1.60).

When we adjusted *P* values for BBPS and sedation, as shown in Table 4 , there were no substantial effects on the results. Differences among centers remained when the same analyses were performed with stratification for each indication.

**Table:** **Table 4** Univariable and multivariable analyses of performance measures after inclusion of BBPS and sedation/analgesia.

		PDR-5			CIR			Severe pain		
Variable	Categories	N (% [95% CI])	*P* value	OR (95 % CI)	N (% [95% CI])	*P* value	OR (95 % CI)	N (% [95% CI])	*P* value	OR (95 % CI)
**Sex**	Women	1959 (24.6 [23.6–25.5])	**< 0.001**	Reference	7694 (97.4 [97.1–97.8])	0.140	Reference	549 (13.1 [12.1–14.1])	**< 0.001**	Reference
	Men	2029 (29.9 [28.8–30.9])		**1.30 (1.20–1.40)**	6584 (97.8 [97.5–98.2])		1.12 (0.85–1.48)	164 (4.6 [3.9–5.2])		**0.46 (0.38–0.56)**
**Age, years**	< 40	280 (11.1 [9.9–12.3])	**< 0.001**	Reference	2465 (98.9 [98.5–99.3])	**< 0.001**	Reference	71 (11.4 [8.9–13.9])	0.290	Reference
	40–49	392 (19.6 [17.8–21.3])		**1.86 (1.56–2.20)**	1969 (98.9 [98.4–99.4])		0.88 (0.46–1.68)	58 (8.2 [6.2–10.3])		0.71 (0.49–1.03)
	50–59	627 (22.8 [21.3–24.4])		**2.27 (1.94–2.65)**	2670 (98.1 [97.5–98.6])		**0.49 (0.28–0.84)**	134 (9.5 [8.0–11.1])		0.87 (0.63–1.20)
	60–69	1198 (35.8 [34.2–37.4])		**3.99 (3.45–4.62)**	3241 (97.5 [97.0–98.1])		**0.46 (0.28–0.77)**	184 (8.5 [7.3–9.7])		0.78 (0.57–1.06)
	70–79	1136 (36.1 [34.4–37.8])		**4.08 (3.52–4.73)**	2988 (96.2 [95.5–96.8])		**0.30 (0.18–0.49)**	210 (9.4 [8.2–10.6])		0.85 (0.63–1.15)
	≥80	355 (35.4 [32.4–38.3])		**4.10 (3.41–4.92)**	945 (95.4 [94.0–96.7])		**0.37 (0.21–0.66)**	56 (8.6 [6.4–10.7])		0.74 (0.50–1.09)
**Indications**	Symptoms	2206 (24.9 [24.0–25.8])	**< 0.001**	Reference	8573 (97.5 [97.1–97.8])	**< 0.001**	Reference	447 (10.1 [9.2–11.0])	**< 0.001**	Reference
	Surveillance (polyp, CRC)	843 (44.9 [42.6–47.1])		**1.90 (1.71–2.12)**	1834 (98.1 [97.5–98.7])		**4.49 (2.95–6.86)**	73 (6.1 [4.7–7.4])		**0.65 (0.50–0.85)**
	CRC family/screening	377 (28.1 [25.7–30.5])		1.12 (0.98–1.28)	1320 (99.0 [98.5–99.6])		1.74 (0.91–3.30)	53 (6.9 [5.1–8.7])		0.77 (0.57–1.05)
	IBD	137 (14.6 [12.3–16.8])		**0.64 (0.52–0.78)**	903 (98.0 [97.2–98.9])		**1.87 (1.03–3.42)**	25 (5.9 [3.6–8.1])		**0.51 (0.33-–0.80)**
	Other	404 (24.5 [22.4–26.6])		**0.79 (0.70–0.89)**	1578 (96.9 [96.1–97.8])		1.06 (0.72–1.56)	112 (12.3 [10.2–14.4])		**1.28 (1.01–1.61)**
	Missing	21 (22.6 [14.1–31.1])		0.93 (0.56–1.53)	70 (88.6 [81.6–95.6])		0.59 (0.22–1.56)	3 (5.7 [0–11.9])		0.56 (0.17–1.84)
**Center**	Center 1	479 (24.2 [22.3–26.1])	**< 0.001**	1.00 (0.88–1.14)	1856 (96.5 [95.6–97.3])	**< 0.001**	**0.54 (0.36–0.79)**	90 (9.7 [7.8–11.7])	**0.040**	0.82 (0.63–1.07)
	Center 2	268 (22.5 [20.1–24.9])		**0.72 (0.62–0.83)**	1116 (95.2 [94.0–96.4])		**0.40 (0.27–0.60)**	78 (11.7 [9.2–14.1])		1.16 (0.89–1.53)
	Center 3	2933 (28.2 [27.3–29.0])		Reference	10228 (98.6 [98.4–98.8])		Reference	467 (8.6 [7.9–9.4])		Reference
	Center 4	308 (25.9 [23.5–28.4])		**0.78 (0.67–0.90)**	1078 (93.0 [91.5–94.5])		**0.33 (0.22–0.48)**	78 (10.1 [8.0–12.3])		**0.71 (0.54–0.93)**
**BBPS**	≥ 6	3637 (26.9 [26.2–27.7])	0.200	Reference	13337 (99.5 [99.4–99.6])	**< 0.001**	Reference	636 (8.9 [8.3–9.6])	**0.009**	Reference
	< 6	171 (30.2 [26.4–34.0])		0.99 (0.82–1.20)	519 (92.5 [90.3–94.7])		**0.07 (0.05–0.11)**	28 (9.6 [6.2–12.9])		1.17 (0.77–1.76)
**Sedation/analgesia**	Yes	1350 (25.2 [24.0–26.3])	**< 0.001**	1.07 (0.98–1.17)	5093 (96.3 [95.8–96.8])	**< 0.001**	**0.67 (0.50–0.90)**	452 (17.1 [15.6–18.5])	**< 0.001**	**3.36 (2.81–4.02)**
	No	2585 (28.1 [27.2–29.0])		Reference	8992 (98.4 [98.2–98.7])		Reference	257 (5.1 [4.5–5.7])		Reference
BBPS, Boston Bowel Preparation Scale; CI, confidence interval; CIR, cecal intubation rate; CRC, colorectal cancer; IBD, inflammatory bowel disease; OR, odds ratio; PDR-5, proportion of at least one polyp ≥ 5 mm in size per colonoscopy. Bold font identifies significant results. Center 1: University clinic. Centers 2 and 4: Local hospitals. Center 3: Outpatient endoscopy practice. *P* values derive from Pearson’s chi-squared test (univariable analysis). ORs were calculated through a binary logistic regression (multivariable analysis).										

## Discussion

We evaluated 14,765 first colonoscopies from western Norway registered in Gastronet over 2 years. There were significant effects from the examined case-mix factors (age, sex, and indication) on the colonoscopy performance measures PDR-5, CIR, and pain.

First, we demonstrated that all factors strongly influence PDR-5. Consistent with previous findings 13 , we found that older patients and men had higher PDR-5. We found that IBD had the lowest PDR-5 and surveillance the highest. These results align with earlier studies 7 19 21 22 . Overall, previous studies have found that ADR in primary screening was comparable to ADR in clinical colonoscopies. A consistent finding in these studies was that the indication surveillance after polypectomy had a high ADR. The indication IBD had a relatively low PDR-5 in our material, even in the age group ≥ 50 years. This is surprising because we have learned that IBD increases risk of colorectal cancer 23 . A recent study from Sweden concluded that there was an increased risk of neoplastic colorectal polyps in IBD patients 24 . The low PDR-5 in IBD patients in our data may be due to several colonoscopies from an early age and surveillance. Also, carcinogenesis in IBD might not follow the traditional adenoma-carcinoma sequence. There may be non-mass-forming dysplasia in IBD, which can be challenging to detect 23 .

CIR was influenced by age and indication. Younger patients had significantly higher CIR, as shown by Nass et al. 9 . CIR was not associated with sex in our data; this differs from the study by Nass et al., which showed that male sex was associated with higher CIR. Good-quality BBPS was significantly associated with high CIR. This concurs with earlier studies, e.g. Hoff et al. 25 . Sedation was associated with higher CIR, which indicates more successful intubation when pain is relieved.

Among indications, surveillance and IBD had higher CIR and less pain. This may be due to repeated colonoscopies and adequate sedation related to previous colonoscopy experiences.

Pain is the only parameter reported by patients in this study. Even though it is an important performance measure, pain is rarely reported in larger colonoscopy studies. To the best of our knowledge, our study is one of the few that have examined patient-reported pain in addition to the performance measures polyp detection and cecal intubation. Sex and indication, but not age, were associated with pain. Age < 40 years was a significant factor for severe pain in the univariable analysis, but this association was no longer significant in the multivariable analysis. This differs from the studies by Seip et al. 14 and Holme et al. 15 . Pain was highly associated with increased sedation/analgesia. This may indicate that when sedation/analgesia is provided, its dosage and timing is inadequate. It may also indicate that other factors besides sedation are essential to reduce pain, e.g. colonoscopy technique.

There were significant differences among the centers in case-mix and performance measures. The difference in withdrawal time between the centers is especially interesting. Center 1 had a longer withdrawal time than the other centers, at 10 minutes, and this may contribute to the relatively high PDR-5 at Center 1, even though they had a large cohort of IBD patients. Center 2 had a relatively low withdrawal time of 7 minutes, which can lead to a lower PDR-5. A limitation is that we only included withdrawal time in colonoscopies during which no interventions or biopsies were performed. This reflects visualization time more adequately than when time is used on procedures, leaving out 63.2% of the colonoscopies. However, 24.8% of the included colonoscopies had withdrawal time < 6 minutes. ESGE performance measures for lower gastrointestinal endoscopy recommend a minimum withdrawal time of 6 minutes and a target of 10 minutes 4 . All centers met the minimum performance standards for PDR-5, CIR, and severe pain. They also performed well on bowel cleansing, with > 90% adequate BBPS. However, there are apparent differences in the performance measures among the centers, which may depend on the individual endoscopist.

A strength of our study is the large number of colonoscopies and inclusion of different clinical indications, which reflect everyday practice. The different types of centers represent the spectrum of outpatient colonoscopy services. Gastronet is widely used in Norway and encompasses a solid database with many parameters.

Retrospective evaluation of data on a center level with few centers may limit general conclusions about center differences. Individual endoscopist factors such as experience, volume, and training may explain some of the center differences. Individual endoscopist data were not available in this study. Gastronet data rely on self-reporting by individual endoscopists, and missing reports may introduce selection bias. Coverage of Gastronet for colonoscopy in the four studied centers was 62% to 94% in 2020 and 2021. Because these data are used to evaluate colonoscopy performance, low coverage represents an important limitation to quality registers. Self-reported data may be incomplete or biased when the same data are used publicly to study the endoscopy centers 26 . Endoscopists may also be prone to the Hawthorne effect, i.e. attempt to change or improve their behavior when being evaluated or studied 27 . A limitation regarding patient experience and pain is a relatively low patient response, varying from 47.3% to 65.8%.

A future aspect of evaluation of colonoscopy quality is a more objective standardization of performance indicators. To adjust the performance measures for case-mix, various methods have been suggested, e.g. the ADR-ESS (ADR Extended to all Screening/ Surveillance) Score by Ladabaum et al. 10 and the observed/expected ratio (O/E ratio) by Nass et al. 9 . This may be considered in quality registries like Gastronet.

In the future, artificial intelligence (AI) may also aid in standardizing performance measures and reporting. Endoscopic imaging is well suited for evaluation by AI, not only as a polyp detection and characterization tool but also as a quality-of-endoscopy tool. AI can evaluate bowel preparation, completeness of colonoscopy, and withdrawal time, parameters that are subjectively reported by endoscopists today. This may influence the performance measure thresholds and warrants future studies.

## Conclusions

This study found that case-mix (age, sex, and indication) significantly influences the colonoscopy performance measures PDR-5, CIR, and severe pain on a center level. This may affect institutional and individual colonoscopy quality parameters, which is vital for the individual track record of endoscopists. Although we showed center differences in performance, other factors, such as individual endoscopist skills, probably influence performance measures. The effect of using case-mix-adjusted quality parameters for evaluation of colonoscopy performance warrants further prospective follow-up studies.
